# Two *Vibrio* species co-colonize a morphologically complex symbiotic light organ

**DOI:** 10.1093/ismejo/wrag063

**Published:** 2026-03-24

**Authors:** Clotilde Bongrand, Raphaël Lami, Marcelino T Suzuki, Eric J Koch

**Affiliations:** Sorbonne Université, UPVD, CNRS, UMR8176 Laboratoire de Biodiversité et Biotechnologies Microbiennes (LBBM), 66650 Banyuls-sur-Mer, France; Sorbonne Université, UPVD, CNRS, UMR8176 Laboratoire de Biodiversité et Biotechnologies Microbiennes (LBBM), 66650 Banyuls-sur-Mer, France; Sorbonne Université, UPVD, CNRS, UMR8176 Laboratoire de Biodiversité et Biotechnologies Microbiennes (LBBM), 66650 Banyuls-sur-Mer, France; Sorbonne Université, UPVD, CNRS, UMR8176 Laboratoire de Biodiversité et Biotechnologies Microbiennes (LBBM), 66650 Banyuls-sur-Mer, France

**Keywords:** symbiosis, squid–vibrio, mutualism, cephalopod, *Vibrio*

## Abstract

The squid–vibrio symbiosis has illuminated fundamental mechanisms of beneficial animal–microbe associations, yet the interactions within sepiolid squid of the Mediterranean Sea remain underexplored. Here, we characterize the *Sepiola affinis* squid–vibrio symbiosis by combining whole-genome sequencing of light-organ isolates, confocal microscopy, and temperature-dependent growth assays. Comparative genomic analyses (ANI, phylogenomics, and functional analyses) revealed two previously undescribed *Vibrio* species to be symbionts of the *S. affinis* light organ. One of the species clusters more distantly from other *Vibrio* species, whereas the second is closer to established *Vibrio* clades and exhibits an expanded repertoire of mobile elements and type VI secretion components, suggesting heightened capacity for genetic exchange and interbacterial interaction. Furthermore, confocal microscopy of juvenile squid established that the *S. affinis* light organ comprises twelve crypts connected by pores and ducts, expanding the number of symbiotic niches relative to other sepiolid squid. In addition, fluorescently labeled isolates from the two *Vibrio* species colonized juveniles in both mono- and co-colonization patterns within crypts. Finally, growth assays across 16°C–24°C identified species-specific temperature differences, indicating temperature preferences that may align with seasonal variability in the Mediterranean Sea. Together, these findings position *S. affinis* as a tractable model for studying how symbiont diversity, organ architecture, and interbacterial interactions contribute to the stability of a mutualistic symbiosis.

## Introduction

Animals and microbes routinely form symbiotic relationships that provide benefits for both partners [1]. These mutualisms are oftentimes integrated into aspects of host physiology, including development, immunity, and nutrition [2]. However, their inherent complexity presents a challenge for understanding the mechanisms underlying their stability. One way of addressing these challenges is through invertebrate models of symbiosis [3]. The partnership between sepiolid squid (i.e. bobtail squid) and bioluminescent *Vibrio (Aliivibrio)* bacteria (i.e. the squid–vibrio symbiosis) has been a model for beneficial symbioses for >30 years [4–6]. Primarily known for research with the Hawaiian bobtail squid *Euprymna scolopes* and its symbiont *Vibrio fischeri*, the symbiosis has yielded insights into how animals and bacteria initially form and subsequently maintain a mutually beneficial relationship. In addition, sepiolid squid occur throughout the world’s oceans, and many of them contain symbiotic associations with bioluminescent bacteria [7].

**Figure 1:**
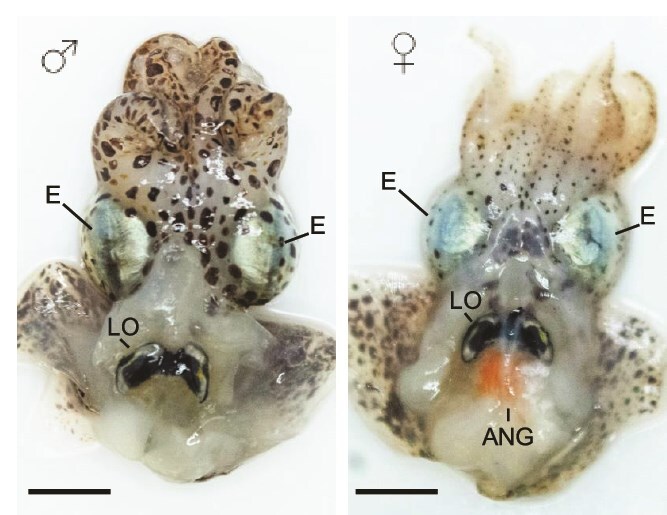
*Sepiola affinis* anatomy. Ventral dissections of a male (left) and a female (right); eyes (E) and light organ (LO) are indicated in both, and the accessory nidamental gland (ANG) is indicated in the female. Scale bars = 0.5 cm. Photographs courtesy of Alice Rodrigues.

The most well-known symbiosis between sepiolid squid and *Vibrio* bacteria occurs within a dedicated light organ (Fig. 1). When the squid first hatch, the light organ is nonsymbiotic and acquires the bacterial symbiont from the surrounding environment [8]. The planktonic *Vibrio* migrate through sets of pores on both sides of the light organ, with each pore connecting to a duct and then ending at an epithelium-lined crypt [9]. The bacteria proliferate within the crypts, establishing symbiont populations that will remain for the lifespan of the squid. Within these crypt spaces, the host provides nutrients to the bacteria in exchange for bioluminescence that is used for counterillumination during nocturnal activity [10]. In both *E. scolopes* and *E. berryi*, the bilaterally symmetrical light organ contains a total of six crypts (i.e. three on each side) and the *Sepiola robusta* light organ has two additional crypts for a total of eight [11–13].

Multiple studies have examined the diversity of bacteria colonizing the sepiolid light organ [14, 15]. In *E. scolopes*, only *V. fischeri* has been found to naturally colonize; however, there is variation at the strain level of bacterial symbionts [16]. In addition to genomic analyses, behavioral diversity among *V. fischeri* strains during colonization has also been characterized [17–19]. Furthermore, similar behaviors during colonization of *E. scolopes* are exhibited in *V. fischeri* strains isolated from other hosts, including the Japanese squid *E. morsei*, suggesting conserved colonization mechanisms [20]. The light organ in *E. berryi* can also be colonized by *V. fischeri* [13]. Interestingly, sepiolid squid from the Mediterranean Sea harbor multiple species of *Vibrio* symbionts within their light organ [21]. Specifically, both *S. robusta* and *S. affinis* are colonized by *V. fischeri* and other *Vibrio* species. The *Vibrio* isolates from *S. affinis* and *S. robusta* also exhibited differences in growth rate according to temperature, with *V. logei* growing more rapidly at colder temperatures relative to *V. fischeri* [21]. Further studies reclassified the species of some of the *Sepiola* light-organ isolates, questioning the presence of *V. logei* and also introducing the species *V. sifiae* [22, 23]*.*

In this study, we examined bacterial species from the *S. affinis* light organ and characterized the morphology of the symbiotic organ, yielding two main findings. First, whole genome sequencing was performed on light-organ symbionts, allowing for the discovery of two uncharacterized species of *Vibrio* bacteria. In addition, *S. affinis* were successfully maintained in the laboratory, providing hatchlings for subsequent colonization with labeled bacterial strains. Colonization with labeled symbionts allowed for characterization of the juvenile light organ morphology, which led to the finding that the *S. affinis* light organ contains a total of 12 crypts. These findings show the greater diversity found within the *S. affinis* light organ relative to other squid light organs and further establish it to be a promising model system to study the squid–vibrio symbiosis. Continuing to explore the *S. affinis* symbiosis can bring unique insights into the evolution and ecology of mutualistic associations.

## Material and methods

### Ethics statement

All live animal collection, husbandry, and experiments were conducted in accordance with Sorbonne Universitie’s and the Centre National de la Recherche Scientifique (CNRS) animal welfare protocols. All maintenance, rearing, and experimental procedures with the squid adhered to the animal welfare standards of EU Directive 2010/63 on the use of animals for scientific purposes. In France, the Observatoire Océanologique has an official ethical commission agreement (A6601601) to ensure that animal welfare standards are maintained.

### *S. affinis* husbandry

Adult *S. affinis* were collected at night during diving expeditions at ~20 m of water depth near Banyuls-sur-Mer, France. The squid were maintained at the Observatoire Océanologique in 30 l aquariums with flowing natural seawater that varied with the seasonal temperatures (e.g. ~13°C in winter to ~24°C in summer). The animals were maintained on a 12-h:12-h light:dark lighting schedule and fed live shrimp every night. Each morning, the aquariums were inspected and any eggs laid were removed, rinsed 3 × 10 min in 0.22 μm filter-sterilized ocean water (FSOW), and then transferred to an aquarium with 5 l of FSOW and continuous aeration. The egg clutches were housed in individual aquariums and underwent a 50% water change every day and maintained at ~23°C. Upon hatching, the squid were removed and placed into fresh FSOW.

### Bacterial isolation

All bacterial strains used in this study were isolated from field-caught *S. affinis* collected during trips in May, June, and July of 2022. To sample the light-organ symbionts, each squid was briefly rinsed 3 × 5 min in FSOW, anesthetized using 2% EtOH in FSOW, and then euthanized by increasing the concentration of EtOH to 4%. The central core tissues from the light organ, which contain the symbiotic crypts, were dissected out, combined for each squid in 70% FSOW diluted with distilled water, and homogenized using a pestle to release the bacteria. Half of the bacteria were immediately mixed with glycerol for a final concentration of 20% and frozen at −80°C, while the remaining homogenate underwent serial dilutions that were then grown on Luria-Bertan salt medium (LBS) agar [24] plates at three temperatures: 4°C, 20°C, and 25°C. Following incubation for 24 h (20°C and 25°C) or 5 days (4°C), ~100 total colonies were selected from each light organ across the temperatures with a focus on selecting bacteria that displayed different sizes or colors. Each colony was transferred from the agar plate to liquid LBS using a toothpick and grown at 25°C with 100 revolutions per minute (RPM) shaking. After 24–48 h, cultures that exhibited visible growth had a subsample removed, to which glycerol was added for a final concentration of 20%, and were frozen at −80°C to create viable frozen stocks.

### Whole genome sequencing

Genomic DNA (gDNA) was extracted from cultures grown in LBS during the exponential phase using the DNeasy blood and tissue kit (QIAgen) according to the manufacturer’s protocol. The quantity and quality of gDNA extracted was measured using a Quantus fluorometer and a Denovix DS11 Spectrophotometer, respectively. Twelve samples (four isolates per light organ from each of three squid) were then sent to Plasmidsaurus (Eugene, OR) for whole genome sequencing using Oxford Nanopore technology in addition to assembly and annotation. All genomes underwent taxonomic analysis using Sourmash v4 and Mash [25, 26]. In addition, the Genome Taxonomy Database GTDP-tk was also used to further characterize the taxonomy of each genome [27, 28]. Average nucleotide identity (ANI) analysis using FastANI v1.34 was performed against reference genomes of the top matches from Sourmash, MASH, and GTDB-tk, in addition to various other *Vibrio* strains [29]. An ANI similarity >95% was used to define the same species, whereas >99.9% ANI similarity indicated the same strain, which were combined for further analyses [29]. Digital DNA–DNA hybridization (dDDH) was performed with the online Genome to Genone Distance Calculator [30, 31]. To construct a phylogenetic tree of the distinct strains used in the ANI analysis, Orthofinder 3.0 was used with default parameters in combination with RAxML-NG [32, 33]. After establishing the taxonomy, a Roary analysis with a 90% identity threshold was used to define genes that were shared or distinct among the newly isolated strains [34]. To compare gene functions, eggNOG-mapper 2.1.12 was used for a Clusters of Orthologous Groups (COG) analysis [35].

### Bacterial strains and labeling

The strains used in this study are listed in Table S1. Cultures were revived from −80°C stocks and grown overnight in LBS medium. For confocal imaging, strains were fluorescently labeled using triparental mating [36] with plasmids pVSV208 (red fluorescent protein) or pVSV102 (green fluorescent protein) [37]. Where appropriate, the medium was supplemented with chloramphenicol (2.5 μg ml^−1^) for pVSV208 or kanamycin (100 μg ml^−1^) for pVSV102.

### Colonization procedure

Bacterial strains were started from −80°C glycerol stocks onto two LBS agar plates and grown overnight at either 20°C or 25°C. Individual colonies were transferred into 5 ml of LBS and grown overnight at 20°C or 25°C with 100 RPM shaking. The following morning, the culture with the highest level of growth determined by optical density at 600 nm (OD_600_) was diluted 1:1000 into seawater-based tryptone (SWT) (700 ml of FSOW, 300 ml H_2_0, 10 g NaCl, 5 g Bacto-Tryptone, and 3 g yeast extract) and incubated at 23°C with 100 RPM shaking and a standard colonization procedure was followed [38]. When the bacteria had reached OD_600_ ~ 0.6, the culture was serially diluted in FSOW 1:10 000 for a final concentration of ~10 000 bacteria ml^−1^ in 150 ml of FSOW. For inoculations with two bacterial strains, each strain was first diluted to an OD_600_ ~ 0.6 and then both added at concentrations of ~5000 CFU ml^−1^, for a total of ~10 000 bacteria ml^−1^. Within 12 h of hatching, *S. affinis* were added to the inoculum and maintained overnight with a water temperature of ~23°C. Following inoculation, the squid were rinsed 3 × 15 min in FSOW and then maintained in FSOW.

### Confocal microscopy

Squid were briefly anesthetized with 2% EtOH in seawater and then fixed with 4% paraformaldehyde in mPBS (50 mM sodium phosphate buffer with 0.45 M NaCl, pH 7.4) for 12 h at 4°C. After fixation, the squid were rinsed 3 × 15 min in mPBS, the mantle opened, and the light organ dissected out and stained for 12 h with TOPRO-3 (Thermofisher Scientific) at a dilution of 1:1000 in mPBS and rhodamine phalloidin (Thermofisher Scientific) at a dilution of 1:40 in mPBS. Samples were rinsed 3 × 20 min at room temperature with gentle rotation and then mounted on glass slides in SlowFade Glass (Thermofisher Scientific). Imaging was performed on a Leica Sp8 Confocal Microscope within the BioPIC platform at the Observatoire Océanologique de Banyuls-sur-Mer. Post-imaging analysis and preparation for publication were performed with FIJI [39], 3D-Slicer [40], and Imaris software (Oxford Instruments).

### Temperature growth assays

Strains were initially grown on LBS agar; then, serial dilutions were grown overnight in LBS, and the dilution at OD_600_ ~ 1.0 for each strain was used further. For growth assays, the equivalent of OD = 0.05 for each strain was used to inoculate 1500 μl SWT and loaded in each well of a 24-well plate. For each strain, three biological replicates (separate cultures grown on different days) were assayed, each with three technical replicates. Plates were incubated with continuous orbital shaking at 16°C, 20°C, or 24°C, and OD_600_ was recorded every 30 min for 10 h and again at 12 h and 24 h on a SpectraMax iD3 (Molecular Devices). To calculate growth rates, OD values were natural-log-transformed, and exponential-phase growth rates were estimated by simple linear regression from 2 to 12 h. Growth rates were compared by two-way analysis of variance (ANOVA) with a Tukey’s multiple-comparisons correction in GraphPad Prism v10.6.0. An adjusted *P* value <0.05 was considered significant.

## Results

### Symbiotic *Vibrio* species from *S. affinis*

**Figure 2:**
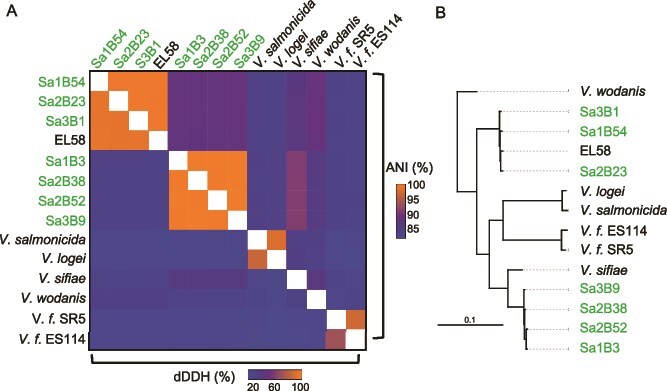
Relatedness of the *S. affinis* isolates. Strains isolated in this study are shown in green. *V. f.* represents *V. fischeri.* (A) Heatmap of average nucleotide identity (ANI; top) and digital DNA–DNA hybridization (dDDH; bottom) among *Vibrio* spp. strains, with colors ranging from higher similarity (orange) to lower similarity (blue). (B) Maximum-likelihood phylogenetic tree inferred from 2096 single-copy *Vibrio* genes using OrthoFinder and RAxML. The scale bar indicates the number of nucleotide substitutions per site.

To examine bacterial diversity within *S. affinis*, 100 strains were isolated from each light organ of three field-caught squid (i.e. 300 total isolates). Selection of the isolates was performed based on colony morphology (e.g. the largest and smallest colonies in addition to colonies with differing pigmentation were chosen) and to provide similar sampling across all three hosts and growth temperatures. During selection, it was observed that many of the colonies were visibly luminescent. The genomic DNA was extracted from twelve total isolates (i.e. four isolates per light organ) and sent for whole genome sequencing. The sequencing results yielded a total of eleven high-quality genomes, with one sample being excluded because of quality issues. Initial ANI analysis to compare each strain revealed that four of the genomes were nearly identical to another sample (ANI > 99.9%), and in these cases, only one strain was used in this study (Fig. 2A). In total, seven distinct strains were obtained from three adults: two strains from each of two adults and three strains from the third adult (Table S1). The genomes of these strains spanned from 4.3 to 4.6 Mb, and all exhibited a GC content of 39%. The seven genomes were grouped into two clusters (consisting of three and four genomes), with 98%–99% ANI similarity, indicating that strains within each grouping represent the same species. Using the definition that >95% ANI similarity represents the same species [29, 41], further analysis against *Vibrio* reference genomes confirmed that the strains are part of the genus *Vibrio* but revealed that none of the isolates were *V. fischeri* nor *V. logei* (Fig. 2A). Furthermore, none of the *Vibrio* (*Aliivibrio*) reference genomes exhibited ANI similarity >95% with the *S. affinis* strains, and the most similar was *V. wodanis* at ~92% ANI similarity. However, the first grouping of three strains (hereafter Banyuls sp. 1) exhibited ANI similarity of ~98% with *Vibrio* sp. strain EL58, indicating that they are from the same species. The bacterial strain EL58 was isolated in 2015 from a gorgonian coral in Faro, Portugal, and identified as an *Aliivibrio* species [42]. The second grouping of four strains (hereafter Banyuls sp. 2) exhibited no ANI similarity >95%, with the highest similarity being with *V. sifiae* (92%). In addition to ANI analysis, dDDH reinforced the finding that the strains belonged to two species that did not share species-level similarity with other known *Vibrio* bacteria (dDDH >70% indicate the same species) (Fig. 2A, bottom). These results led to the conclusion that the newly isolated strains from the *S. affinis* light organ belong to two separate novel *Vibrio* species.

To further investigate the similarity of the *S. affinis* strains relative to other *Vibrio* species, Orthofinder in combination with RAxML-NG was used to construct a phylogenetic tree from single-copy orthologs present in all of the genomes (Fig. 2B) [32, 33]. Consistent with the ANI analysis, Banyuls sp. 1 clustered closely with EL58, and the clade separated themselves from the reference genomes, providing evidence of being from a distinct *Vibrio* species. In contrast, Banyuls sp. 2 clustered more closely to the *Vibrio* reference strains, with the closest being *Vibrio sifiae*, a bioluminescent bacterium originally isolated from surface seawater in Tokyo Bay, Japan [43]. Although all strains within Banyuls sp. 2 clustered tightly together, their positioning among the reference genomes indicates that the species is more similar genomically to the other *Vibrio* spp. than Banyuls sp. 1. Overall, these results show that the bacterial strains isolated from the *S. affinis* light organ for this study represent two previously undescribed species of *Vibrio* that share a symbiotic lifestyle.

### Comparative analyses of the *Vibrio* species

**Figure 3:**
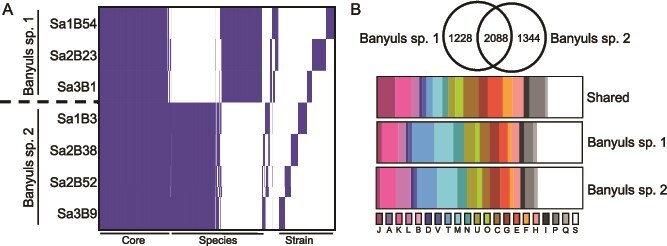
Comparison of two *Vibrio* spp. co-occurring in the *S. affinis* light organ. (A) Roary presence/absence matrix for genes across the seven newly isolated symbiont strains. The two species have 2088 shared genes (Core), ~1300 species-specific genes (Species), and 161–411 strain-specific genes (Strain). (B) Distribution of Clusters of Orthologous Groups (COG) assignments for genes from the Roary analysis. Top: comparison of COG distributions between the two *Vibrio* spp.; bottom: distribution by COG category. COG categories: J: Translation, ribosomal structure and biogenesis; A: RNA processing and modification; K: Transcription; L: Replication, recombination and repair; B: Chromatin structure and dynamics; D: Cell cycle control, cell division, chromosome partitioning; M: Cell wall/membrane/envelope biogenesis; V: Defense mechanisms; N: Cell motility; T: Signal transduction mechanisms; U: Intracellular trafficking, secretion, and vesicular transport; O: Posttranslational modification, protein turnover, chaperones; C: Energy production and conversion; G: Carbohydrate transport and metabolism; E: Amino acid transport and metabolism; F: Nucleotide transport and metabolism; H: Coenzyme transport and metabolism; I: Lipid transport and metabolism; P: Inorganic ion transport and metabolism; Q: Secondary metabolites biosynthesis, transport and catabolism; S: Function unknown.

To further characterize the two *Vibrio* species, a Roary analysis was performed to compare gene presence and absence (Fig. 3). Because of the ~90% ANI similarity between the two species, we lowered the identity threshold for a shared gene from the default of 95%–90%, which has been demonstrated in other studies that examine across different species [44]. The seven genomes each contained 3913–4274 genes. From all of these genes, the Roary analysis built a pangenome consisting of 8107 total genes (Fig. 3A). Within the pangenome, all seven strains shared 2088 genes (~26%), which constituted the core genome. Between the two *Vibrio* species, there were 1228 (~15%) and 1344 (~17%) genes specific to Banyuls sp. 1 and Banyuls sp. 2, respectively. Among the individual strains, there were, on average, 196–455 (~2%–6%) singletons or strain-specific genes.

After establishing patterns of gene presence and absence among the two *Vibrio* species, a COG analysis by EggNOG was performed to compare functionality of the core and species-specific genes (Fig. 3B). Among the core genes, the COG categories J (Translation, ribosomal structure and biogenesis), C (Energy production and conversion), E (Amino acid transport and metabolism), H (Coenzyme transport and metabolism), and P (Inorganic ion transport and metabolism) are prevalent. This is expected because these categories represent fundamental microbial processes that are highly conserved. Among the species-specific genes, the most represented COG categories were T (Signal transduction mechanisms), M (Cell wall/membrane/envelope biogenesis), N (Cell motility), and U (Intracellular trafficking, secretion, and vesicular transport)*.* Furthermore, Banyuls sp. 2 exhibited 180 genes belonging to L (Replication, recombination, and repair) compared to 129 genes in Banyuls sp. 1*.* Among these L-category genes in Banyuls sp. 2, there were numerous genes related to transposase activity and group II introns. The prevalence of transposase and group II intron genes indicates elevated mobile-element activity, consistent with high genetic transfer and a capacity to diversify its genome [45]. In addition, although not consistently found in the same COG category, Banyuls sp. 2 harbors a larger type VI secretion system (T6SS) repertoire: 36 T6SS-associated genes are present in all four Banyuls sp. 2 genomes, whereas only strain Sa2B23 from Banyuls sp. 1 encodes 13 T6SS genes and the other two strains have no annotated T6SS components. These results suggest that Banyuls sp. 2 has higher potential for bacterial cell–cell communication and DNA exchange relative to Banyuls sp. 1.

### Influence of temperature on *Vibrio* species growth

**Figure 4:**
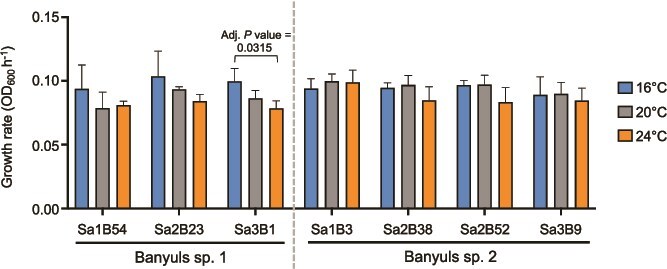
Effect of temperature on growth of *Vibrio* sp*.* strains. Growth rate for each strain measured at 16°C, 20°C, and 24°C. Statistical significance of temperature effects within strains was tested by two-way ANOVA with multiple comparisons; brackets above indicate statistical significance between two growth rates. Error bars denote SD (*n* = 3).

With the knowledge that bacterial strains isolated from the light organs of *Sepiola* spp. exhibit fluctuations in growth rate according to temperature, it was next tested whether the newly isolated *Vibrio* spp. exhibit similar patterns [21]. The growth rates for all seven strains were tested at 16°C, 20°C, and 24°C to simulate cold, medium, and warm temperatures, respectively (Fig. 4, Supplementary Figs S1 and S2). The growth rates for all strains during exponential growth were compared using a two-way ANOVA, and it was found that although the strains were not significantly different from one another (*P* value = .1380), temperature was a significant factor (*P* value = .0037). In addition, the strains belonging to Banyuls sp. 1 exhibited their highest growth rate at 16°C and lowest at 24°C, indicating that the strains grow faster at the colder temperatures (i.e. 16°C > 20°C > 24°C). Only Sa3B1 exhibited statistical significance between growth at 16°C and 24°C (adj. *P* value <.05). For Banyuls sp. 2, the strain growth rates were highest at 20°C, but there was variability with the other temperatures, with Sa1B3 growing slowest at 16°C but the other three strains slowest at 24°C. None of these differences in growth rate were statistically significant when comparing temperatures (adj. *P* value >.05). These results indicate that the newly-isolated *Vibrio* spp. exhibit subtle, yet consistent temperature-dependent differences in growth rate.

### Morphology of the juvenile *S. affinis* light organ

**Figure 5:**
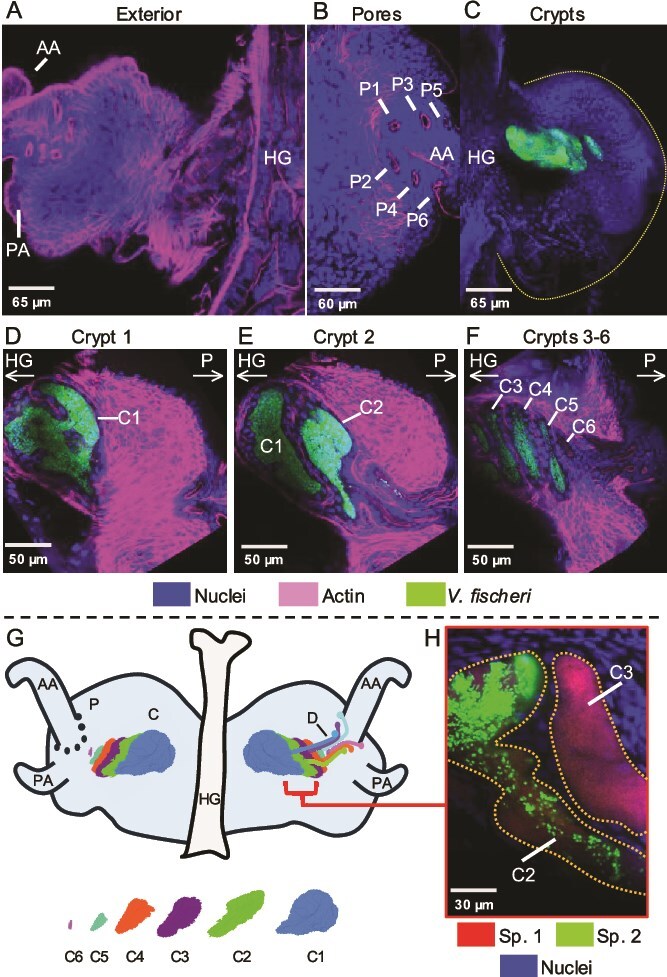
Juvenile light organ of *S. affinis* at 48 h postcolonization. Top row: (A) exterior view showing anterior (AA) and posterior appendages (PA) and hindgut (HG); (B) interior view with symbiont-containing crypts and the light-organ outline (dashed line); (C) pores (P1–P6). Middle row: (D) crypt 1 (C1), (E) crypt 2 (C2), (F) and crypts 3–6 (C3–C6); the arrow indicates hindgut direction. Bottom row: (G) schematic of pores and crypts (left side), with ducts (right side), and individual crypts (below); (H) a representative example of a light organ showing both co-colonized (C2) and mono-colonized (C3) crypts containing newly isolated *Vibrio* spp*.* with Banyuls sp. 1 (Sp. 1) and Banyuls sp. 2 (Sp. 2).

The exterior appearance of the juvenile *S. affinis* light organ is consistent with that of other sepiolid squids [11, 12]. The *S. affinis* light organ is bilobed with a heart-shaped appearance and contains two appendages on each side: a longer anterior appendage and a shorter poster appendage (Fig. 5A). At the base of each anterior appendage, a set of six pores occur that lead inside the light organ (Fig. 5B). The presence of twelve pores per light organ is more than those present in *E. scolopes* and *E. berryi*, which have six pores, or *S. robusta*, which has eight [9, 12, 13]. Each pore leads to a separate duct (Supplementary Fig. S3 that terminates by connecting into a crypt for a total of 12 distinct crypt spaces that will house the bacterial symbionts (Supplementary Fig. S4). To visualize the interior of the juvenile squid colonized by symbionts, *S. affinis* were exposed to fluorescently labeled ES114, a well-studied *V. fischeri* strain that has stability harboring fluorescent plasmids (Fig. 5C) [46, 47]. Within the *S. affinis* light organ, the crypts are arranged in an overlapping formation that makes distinguishing them challenging (Fig. 5C, Movie S1). The largest crypt (Crypt 1) is the most medial and closest to the ventral surface (Fig. 5D). Crypt 2 is slightly smaller, more lateral, and more dorsal than Crypt 1 (Fig. 5E), a successive pattern continued by Crypts 3–6 (Fig. 5F). Crypt 6 is the smallest, most lateral (i.e. closest to the pores and appendages), and most dorsal but was often partially or not colonized. The six crypts create an overall architecture that is highly complex, with overlapping symbiotic niches that are partially stacked and ducts passing over more dorsal crypts (Fig. 5G). The stacking of the crypts allows for a near-continuous population of luminescent bacteria on each side of the light organ that is angled toward the ventral and lateral surfaces.

After initial characterization of the *S. affinis* light organ with ES114, some of the strains isolated from *S. affinis* were labeled with a fluorescent plasmid and tested for colonization. Through confocal microscopy, it was confirmed that Banyuls sp. 1 and Banyuls sp. 2 strains colonize juvenile *S. affinis* when inoculated in pairs originating from the same host (Fig. 5F). Furthermore, the *Vibrio* spp. were observed both in monocolonization and co-colonization of crypt spaces. Although limited to visual examination through confocal microscopy, these results provide evidence that the newly isolated Banyuls sp. 1 and Banyuls sp. 2 strains colonize the *S. affinis* light organ and exhibit colonization patterns (e.g. both mono and co-colonized crypts) similar to how *V. fischeri* strains colonize *E. scolopes* [19].

## Discussion

Natural symbioses examined under laboratory conditions deepen the understanding of how mutualistic associations operate within an ecological context [48, 49]. The squid–vibrio symbiosis is a versatile model for natural animal–microbe mutualisms because it combines a host that can be maintained under both symbiotic and nonsymbiotic conditions with a bacterial partner that is highly tractable in the laboratory [50]. In addition, the study of field-caught *E. scolopes* and *V. fischeri* in their native habitat of Hawaii has allowed for incorporation of ecological factors to provide a well-rounded perspective of environmentally transmitted symbioses [15, 17]. The Mediterranean Sea harbors a wide diversity of sepiolid squid, providing a unique reservoir of squid–vibrio symbioses [7, 51]. Furthermore, with multiple bacterial symbionts, the squid–vibrio symbiosis in *S. affinis* offers a valuable perspective into how bacterial species co-colonize and persist within a symbiotic organ. To better understand how the squid–vibrio symbiosis functions with multiple bacterial species, this study aimed to assess the microbial diversity and light-organ morphology within the sepiolid squid *S. affinis*.

From the *S. affinis* light organ, two species of symbiotic *Vibrio* bacteria were isolated and their genomes characterized. The discovery of these species highlights the value of whole-genome approaches in bacteria, especially in *Vibrio* species. The finding of multiple *Vibrio* species from *S. affinis* light organs was expected because this was found in 1998 using 16S rRNA gene sequences and the species were identified to be *V. fischeri* and *V. logei* [21]. Subsequently, a study using a Multilocus Sequence Analysis (MLSA) approach confirmed the species identity of some *Sepiola* light-organ isolates and reclassified others [23]. Other studies have used different combinations of genes and genomes to examine the *Vibrio* taxonomy, highlighting the complexity of the genus [28, 52]. In particular, one study included the genome of *Vibrio* sp. strain EL58 isolated from a Mediterranean Sea coral and grouped it with *Sepiola* spp. light-organ strains, indicating that these strains represent a *Vibrio* species that exhibits a conserved role of a bacterial symbiont [52].

The presence of both species within each light organ examined is a consistent pattern across three different hosts. Multiple *Vibrio* species within *S. affinis and S. robusta* light organs have been linked to temperature-dependent growth patterns that were exhibited by each *Vibrio* species [21]. In contrast, *E. scolopes* is only colonized in its native habitat of Hawaii by *V. fischeri.* Therefore, the presence of two *Vibrio* species with different temperature preferences within *S. affinis* and *S. robusta* may be an adaptation to the larger seasonal temperature fluctuations in the northwestern Mediterranean Sea relative to the tropical waters surrounding Hawaii. However, the mechanisms of how the host is consistently colonized by multiple species remain unknown. Further study of the newly characterized light-organ symbionts can advance our understanding of partner choice in different environments with temperature variations, providing an intriguing contrast to tropical symbioses with more constant conditions. Moreover, the *S. affinis* squid–vibrio symbiosis provides a tractable system to investigate how climate change, specifically effects related to temperature increases, could reshape marine host–microbe interactions.

The genomes of the seven *Vibrio* strains each exhibit ~4000 genes, which is typical among *Vibrios*. Consistent within the *Vibrio* genus, strain-level diversity appears to be driven by horizontal gene transfer and gene presence/absence variation [53–55], a phenomenon also observed in other bacterial species present in horizontally acquired microbiomes [56, 57]. The functional analysis of the genomes showed that Banyuls sp. 2 contained many genes related to the exchange of genetic material (e.g. transposases and group II intron family genes). Previous work has shown group II intron genes in the pathogenic *Vibrios V. cholerae* and *Vibrio parahaemolyticus* [58], but here, we show the presence of the genes in a light-organ symbiont. In addition, the higher prevalence of T6SS genes across all four Banyuls sp. 2 strains, relative to Banyuls sp. 1, suggests an enhanced capacity for interbacterial interaction in the former. Finally, the phylogenetic analysis provides further support for differences in genomic composition between the two species (Fig. 2B). In the phylogenetic tree, Banyuls sp. 2 clusters more closely with other *Vibrio* spp., indicating higher similarity, whereas Banyuls sp. 1 is comparatively isolated. The higher similarity of Banyuls sp. 2 to the other *Vibrio* spp. may reflect increased horizontal gene transfer (e.g. through transposases and group II intron family genes), whereas Banyuls sp. 1 appears more conserved. Although this study provides an initial characterization of these symbiotic species, additional strains, symbiotic or planktonic, need to be collected and sequenced to better capture the diversity of the species. Furthermore, monitoring of the two species within the light organ across host maturation and seasons would yield key insights into their colonization dynamics and stability.

To visualize the symbiosis within the *S. affinis* light organ, the two *Vibrio* species were labeled with plasmids expressing a green or red fluorescent protein (i.e. GFP or RFP). Strains of both species were able to be successfully transformed with the plasmids, which is a reflection of the compatibility and plasticity of these *Vibrio* species. When visualizing the juvenile light organs of *S. affinis*, the general structure is similar to *E. scolopes, E. berryi*, and *S. robusta* with a bilobed organ that has ciliated appendages and a migration pathway consisting of pores leading to ducts that terminate at crypts [9, 12, 13]. However, the *S. affinis* light organ has 12 crypts, pores, and ducts, more than the 6–8 present in the other squid–vibrio symbioses that have been described. The presence of nearly twice the crypts provides more symbiotic niches for distinct populations of symbionts to grow. In *E. scolopes*, crypts are colonized by a single strain most of the time and the presence of twice the crypts would provide opportunity for increased symbiont diversity. Thus, the greater number of crypts within the *S. affinis* light organ could be an adaptation to promote symbiont diversity and provide greater stability through seasonal fluctuations in the Mediterranean Sea.

Upon examination of the newly characterized *Vibrio* strains within the *S. affinis* light organ, both monocolonized crypts and co-colonized crypts were observed. These patterns of light-organ colonization are similar to those in *E. scolopes*, where *V. fischeri* strains can co-colonize the same crypt or occupy different crypts [17, 18]. However, the number of light organs examined was too low for quantitative analysis. Another difference within the *S. affinis* light organ was the lack of clear antechambers in the ducts and bottlenecks at the entrance to the crypts (Movie S1), which are distinct features of both *E. scolopes* and *E. berryi*. In *E. scolopes*, the bottleneck has been linked to the daily venting behavior that occurs at dawn [59]. However, the lack of a large diel venting cycle in the *S. affinis* light organ may be the reason for the absence of the bottleneck, without a need to suddenly release the majority of the bacterial population. In the bioluminescent Japanese Pinecone Fish *Moncentris japonica*, a similar pattern of slow, continuous release of the bacterial symbionts with random larger pulses led the authors to conclude that the bacterial populations grow continuously [60]. Future studies of the native bacterial symbionts in *S. affinis* will provide evidence if there are species-dependent patterns within the light-organ crypts. Sampling more squid and sequencing more bacterial isolates will shed light on this symbiosis and enable further conclusions on the microbial diversity present in *S. affinis*.

In this study, we deliver an in-depth characterization of the *S. affinis* squid–vibrio symbiosis by examining the light-organ architecture and performing whole-genome analyses of bacterial symbionts. Whole-genome sequencing revealed two previously undescribed *Vibrio* species within this symbiosis. Moreover, genome analyses indicate that one lineage is comparatively distinct relative to other *Vibrio* spp., whereas the second species clusters within prominent *Vibrio* clades and exhibits an expanded repertoire for genetic exchange and T6SS, implying heightened interbacterial interaction. In parallel, we characterized the juvenile *S. affinis* light-organ morphology, finding conserved migration pathways (pores→ducts→crypts) similar to other squid light organs, yet an expanded number of crypts with 12. Taken together, these results support the hypothesis that the *S. affinis* light-organ symbiosis prioritizes symbiont diversity via 12 symbiotic niches and the presence of multiple symbiotic *Vibrio* species.

The discovery of two novel *Vibrio* species provides an initial window into the light-organ symbiosis of *S. affinis*, though further experimentation is needed for a more thorough understanding of the association. In particular, the diversity of *Vibrio* within the association, both at the species and strain level, provides a promising avenue for future investigations. Furthermore, sampling additional squid is essential for understanding how conserved *Vibrio* symbiont diversity is within the *S. affinis* light organ. Because this study examined a subset of the total isolates, the remaining bacteria collected from the three *S. affinis* offer an opportunity to estimate the abundance of each *Vibrio* species and strain within a light organ and potentially identify additional symbionts. In addition, resequencing of light-organ isolates collected from previous studies [21] and comparison with the findings presented here would provide further insight into the diversity of symbiotic *Vibrio*. Notably, all *S. affinis* light organs from this and previous studies [21] were collected during warm weather months due to the increased difficulty in collecting the squid during cold weather. The establishment of a squid husbandry system will allow for monitoring of *Vibrio* dynamics within the light organ through seasonal changes. In addition, this study was limited to squid collected near Banyuls-sur-Mer and expanding collection across the Mediterranean Sea would clarify the prevalence of the two newly characterized species and may reveal additional novel ones. Finally, the broad diversity of symbiotic squid native to the Mediterranean Sea presents an opportunity to examine host–symbiont specificity and determine whether these two *Vibrio* species are present in the light organs of other hosts. In particular, examination of *S. robusta*, native to Banyuls-sur-Mer, offers an intriguing comparison to *S. affinis* because they are sympatric species containing a light organ.

## Supplementary Material

wrag063_Supplementary_materials

wrag063_Supplementary_materials

## Data Availability

The genomes used in this study have been deposited into a public online repository. The data can be found at https://www.ncbi.nlm.nih.gov/ with project number PRJNA1414886 and accession numbers JBTZRY000000000, JBTZRX000000000, JBTZRW000000000, JBTZRV000000000, JBTZRU000000000, JBTZRT000000000, and JBTZRS000000000.
