# Supplementary material for: Two Vibrio species co-colonize a morphologically complex symbiotic light organ
Source: ISME J. 2026 Mar 24;20(1):wrag063. doi: 10.1093/ismejo/wrag063 (PMC13122630; doi:10.1093/ismejo/wrag063)
Supplement: wrag063_Supplementary_materials [file wrag063_supplementary_materials.pdf]

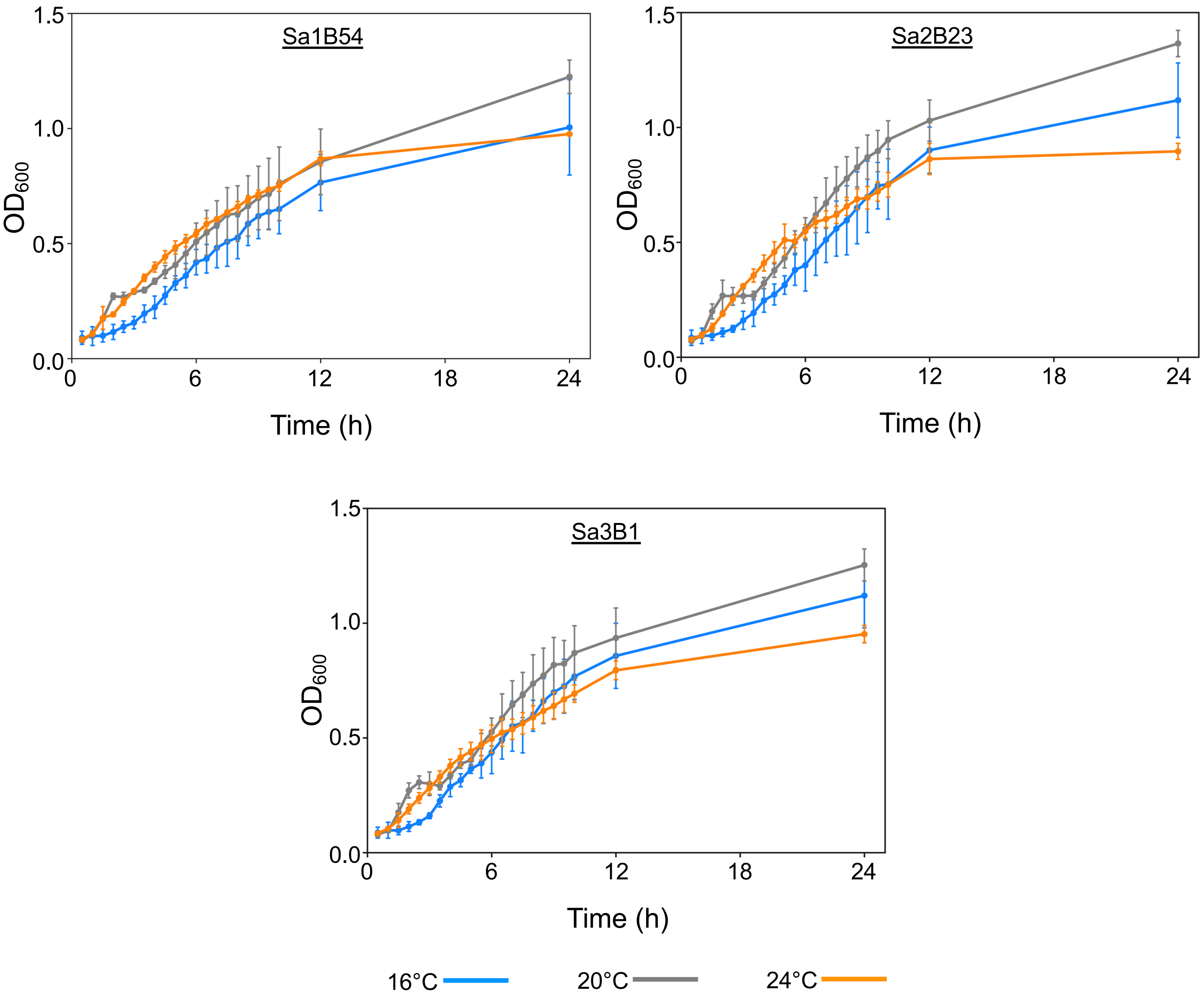

Figure S1. Growth curves for *Vibrio* sp. 1 strains at three different temperatures. The OD_{600} for each strain measured at 16°C (blue), 20°C (grey), and 24°C (orange). Error bars denote SD (n = 3).

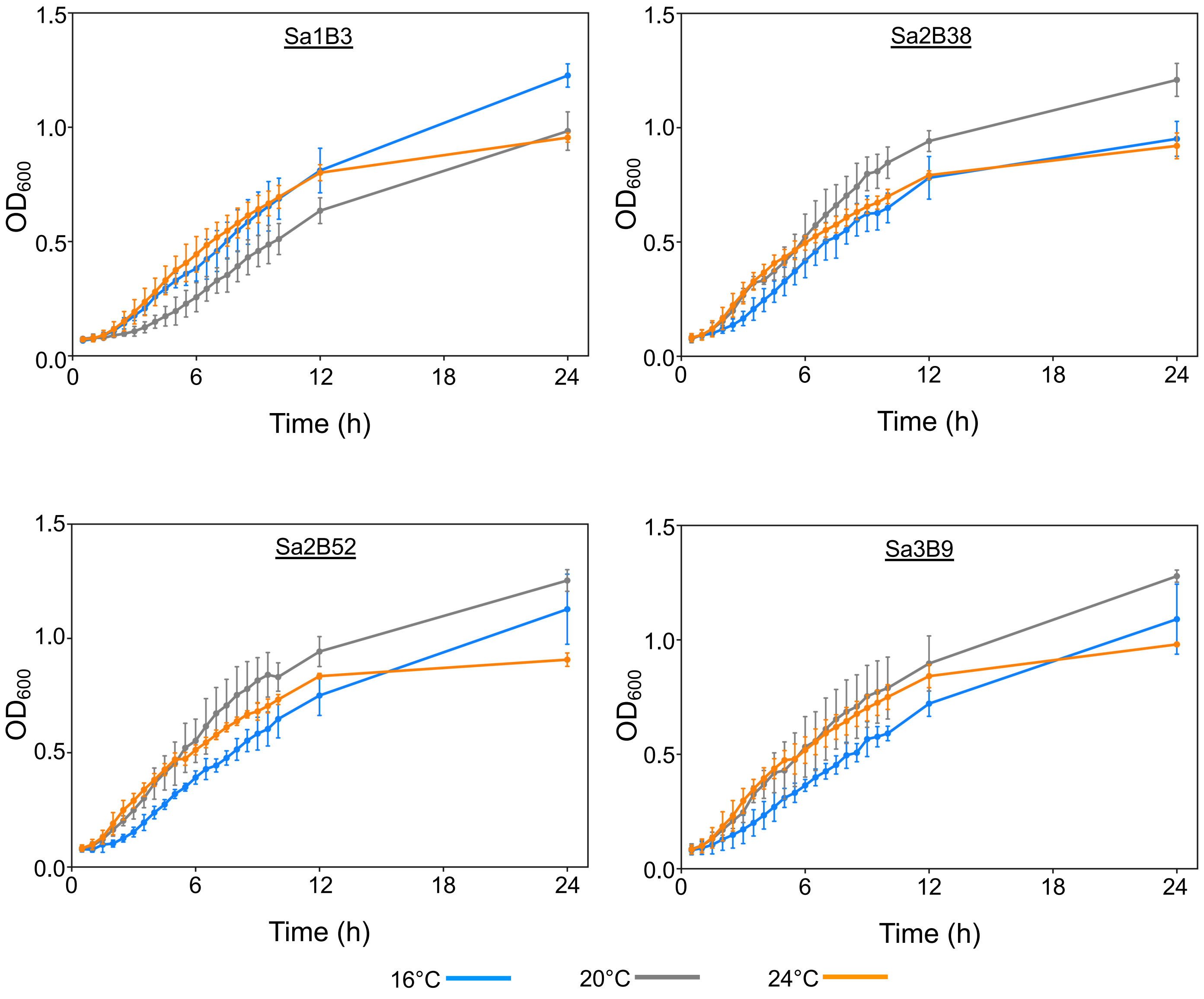

Figure S2. Growth curves for *Vibrio* sp. 2 strains at three different temperatures. The OD_{600} for each strain measured at 16°C (blue), 20°C (grey), and 24°C (orange). Error bars denote SD (n = 3).

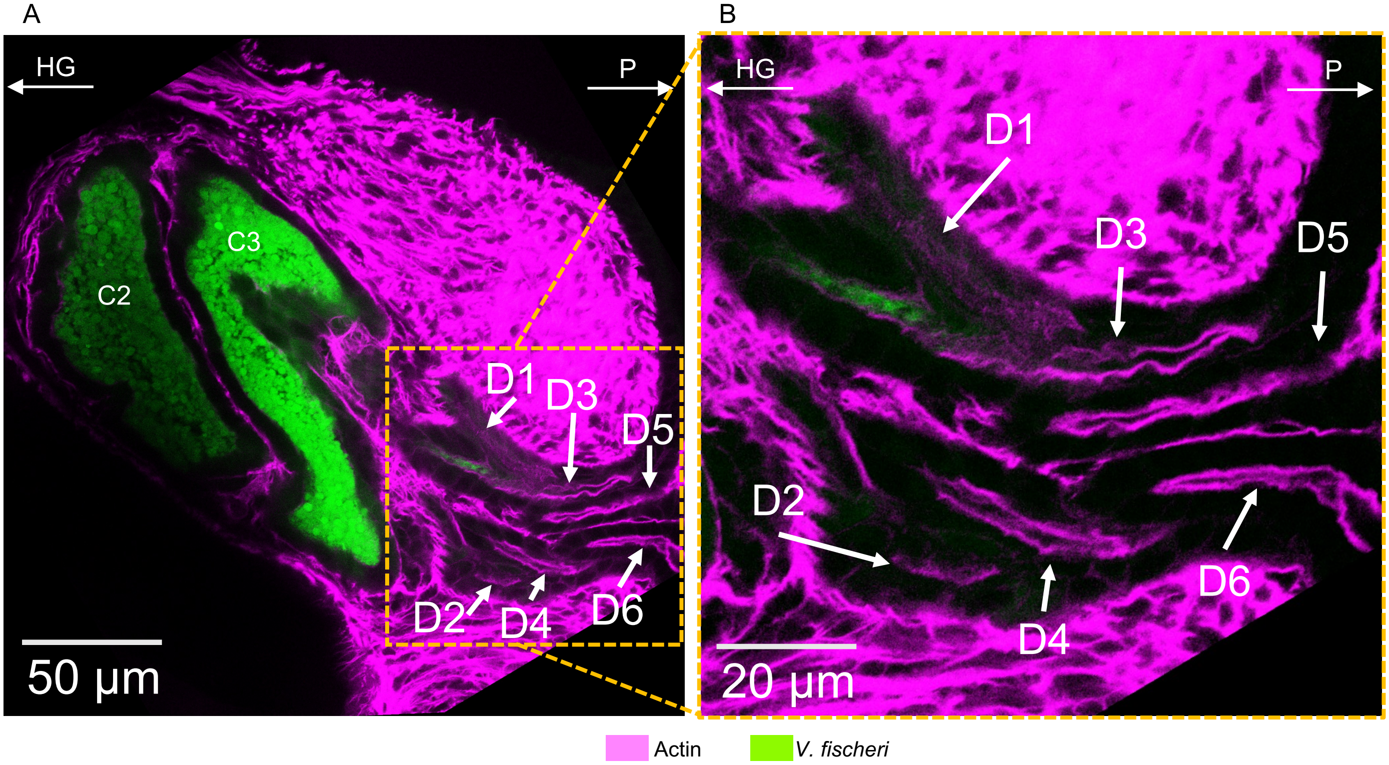

Figure S3. The ducts leading to symbiont-containing crypts. A symbiotic light organ at 48 h post-colonization with the six ducts at *(A)* low and *(B)* high magnification. The ducts (D1-D6) are each indicated with arrows in image B. Crypt 2 (C2) and crypt 3 (C3) are visible in image A. For orientation, the direction of the hindgut (HG) and pores (P) are indicated at the top of each image.

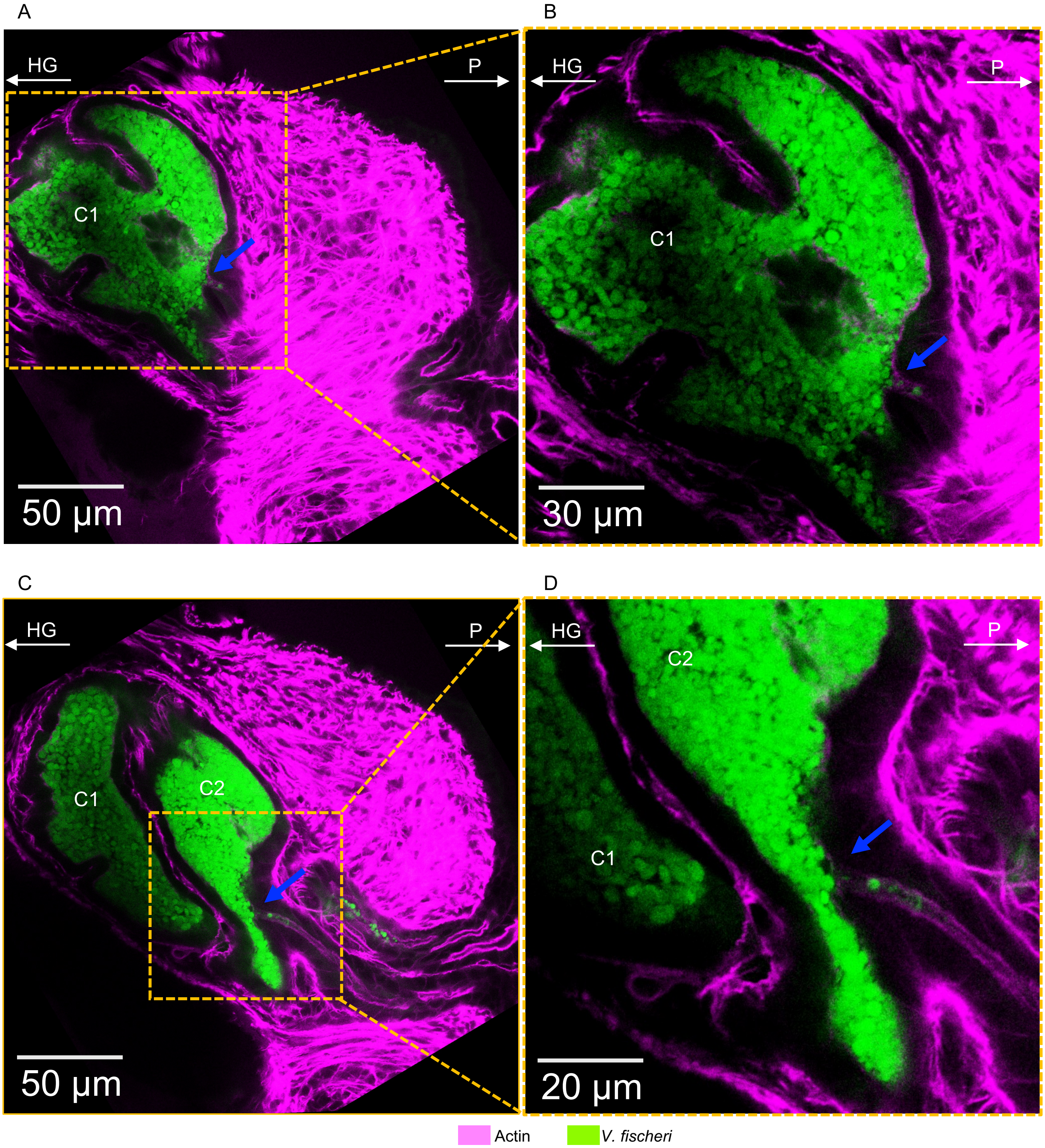

Figure S4. Entrances to symbiont-containing crypts in *S. affinis* at 48 h post-colonization. Top row: Crypt 1 (C1) at *(A)* low and *(B)* high magnification. Bottom row: Crypt 2 (C2) at *(C)* low and *(D)* high magnification. Blue arrows indicate where the ducts connect to the crypts. For orientation, the direction of the hindgut (HG) and pores (P) are indicated at the top of each image.
