# Supplementary material for: Two Vibrio species co-colonize a morphologically complex symbiotic light organ
Source: ISME J. 2026 Mar 24;20(1):wrag063. doi: 10.1093/ismejo/wrag063 (PMC13122630; doi:10.1093/ismejo/wrag063)
Supplement: wrag063_Supplementary_materials [file wrag063_supplementary_materials.zip › Supplementary_materials_wrag063_Table_S1.docx]

| Strain name | Assembly  accession number | Genome size (Mb) | Number of proteins | % GC | Host |
| --- | --- | --- | --- | --- | --- |
| *Vibrio* sp. Sa1B3 | JBTZRY000000000 | *4.6* | *4274* | 39% | *Sepiola affinis* |
| *Vibrio* sp. Sa1B54 | JBTZRX000000000 | *4.5* | *4080* | 39% | *Sepiola affinis* |
| *Vibrio* sp. Sa2B23 | JBTZRW000000000 | *4.6* | *4220* | 39% | *Sepiola affinis* |
| *Vibrio* sp. Sa2B38 | JBTZRV000000000 | *4.6* | *4188* | 39% | *Sepiola affinis* |
| *Vibrio* sp*.* Sa2B52 | JBTZRU000000000 | *4.5* | *4181* | 39% | *Sepiola affinis* |
| *Vibrio* sp*.* Sa3B1 | JBTZRT000000000 | *4.3* | *3913* | 39% | *Sepiola affinis* |
| *Vibrio* sp*.* Sa3B9 | JBTZRS000000000 | *4.5* | *4113* | 39% | *Sepiola affinis* |
| *Vibrio* sp*.* EL58 | GCF_900312675.1 | *4.3* | *3855* | 39% | *Eunicella labiata* |
| *V. salmonicida* LFI1238 | GCF_000196495.1 | *4.6* | *4286* | *41%* | *Gadhus morhua* |
| *V. logei* 1S159 | GCF_001691055.1 | *4.6* | *4111* | *39%* | *N/A* |
| *V. sifiae* NBRC 105001 | GCF_002954715.1 | *4.7* | *4175* | *38%* | *N/A* |
| *V. wodanis* BL6 | GCF_052920805.1 | *5.2* | *4246* | 38.5% | *Salmo salar* |
| *V. fischeri* SR5 | GCF_000241785.1 | *4.3* | *3810* | 38% | *Sepiola robusta* |
| *V. fischeri* ES114 | GCF_000011805.1 | *4.3* | *3819* | *38%* | *Euprymna scolopes* |

**Table S1**. Strains used in the study.
